# Phosphoinositide metabolism-related prognostic genes and their prognostic value in cervical cancer: an investigation based on transcriptome, single-cell analyses and verification

**DOI:** 10.1186/s12885-026-16509-x

**Published:** 2026-07-09

**Authors:** Hui Meng, Li Tan, Ying Chen, Dongmei Wang, Yong Qi, Chuchao Zhu

**Affiliations:** 1 Department of Transfusion, General Hospital of Central Theater Command, Wuhan, 430070 Hubei China; 2 Department of Disease Control and Prevention, General Hospital of Central Theater Command, Wuhan, 430070 Hubei China; 3 Department of Obstetrics and Gynecology, General Hospital of Central Theater Command, Wuhan, 430070 Hubei China

**Keywords:** Cervical cancer, Phosphoinositide metabolism, Bioinformatics, Prognostic gene

## Abstract

**Background:**

Cervical cancer (CESC) is a major gynecological malignancy, and abnormalities in phosphoinositide metabolism (PPM) are closely linked to tumor progression. This study aimed to screen phosphoinositide metabolism-related genes (PPM-RGs) with prognostic value for CESC and explore their molecular mechanisms and clinical significance.

**Methods:**

CESC transcriptomic datasets and PPM-RGs were retrieved from public databases. Differentially expressed genes were intersected with PPM-RGs to obtain candidate genes. Prognostic genes were screened via univariate Cox regression and proportional hazards assumption test, and a risk model was constructed using random survival forest (RSF). Independent prognostic analysis and nomogram establishment were performed, followed by pathway enrichment, immune infiltration, somatic mutation and single-cell analyses to characterize the tumor microenvironment. Immunohistochemical staining was used to verify the protein expression of key genes in CESC tissues.

**Results:**

Twenty candidate genes were initially identified, and two protective prognostic genes VAC14 and INPP5K were finally determined. The risk model showed favorable predictive performance with area under the curve (AUC) over 0.7, and high-risk patients had poorer survival. The nomogram combining risk score and M stage presented high accuracy. The high-risk group exhibited distinct pathway enrichment, altered immune infiltration and low somatic mutation rates. Monocytes were identified as potential key cells with impaired communication and differentiation in CESC. Immunohistochemistry confirmed downregulated expression of the two genes in CESC epithelial cells.

**Conclusion:**

VAC14 and INPP5K were PPM-related prognostic genes for CESC. Monocytes played critical roles in CESC progression. The risk model and nomogram aided prognosis prediction, providing new insights for CESC treatment.

**Supplementary Information:**

The online version contains supplementary material available at https://doi.org/10.1186/s12885-026-16509-x.

## Background

Cervical cancer (CESC) is the fourth leading malignant tumor among women globally. In 2022, around 662,000 new cases and 349,000 deaths were recorded worldwide, with about 90% of these occurring in developing countries [1]. Compared with most other malignant tumors, cervical cancer demonstrates an earlier age of onset, thereby posing substantial risks to female reproductive function [2]. Despite the effectiveness of HPV vaccination, regular screening, and early treatment can effectively prevent the majority of cervical cancer cases [3, 4], mortality rates continue to rise, particularly in resource-limited regions [1, 5]. Current clinical interventions primarily include surgery, radiotherapy, chemotherapy, and immunotherapy, with specific strategies tailored according to the cancer stage [5]. However, these approaches have provided only limited improvement in patients’ overall survival. Therefore, there is a pressing need to develop more accurate prognostic models and effective therapeutic targets. Recently, rising interest has been given to the roles of the tumor microenvironment and metabolic reprogramming in the progression of cervical cancer [6–8]. Research indicates that metabolism‑immune interactions play a pivotal role in CESC development. In cervical cancer, IFN-γ-producing Th1 cells are associated with a better prognosis, while the polarization of Th2 and Th17 cells correlates with disease progression. Lipid metabolic reprogramming reshapes the profile of bioactive lipids, thereby not only fueling the rapid proliferation of tumor cells but also inducing metabolic exhaustion in tumor-infiltrating T cells, thereby further impairing the host’s antitumor immune response [8]. Therefore, elucidating the cross-regulatory mechanisms between metabolism and immunity is of great significance for constructing precise prognostic models, developing combination therapies, and improving patient outcomes.

Phosphoinositides are lipid signaling molecules derived from phosphatidylinositol that function as central integrators of membrane dynamics, with profound impacts on cellular physiology and disease pathogenesis [9, 10]. In recent years, the roles of genes involved in phosphoinositide metabolism (PPM) in human cancers have been progressively elucidated. As a critical intracellular signaling transduction pathway, a pivotal role has been attributed to the PI3K-Akt signaling pathway in suppressing apoptosis and driving cell proliferation by regulating the activation profiles of diverse downstream effectors. It is closely associated with tumor initiation, progression, and drug resistance [11–13]. In CESC, PPM is markedly dysregulated. The canonical phosphoinositide phosphatase PTEN functions as a critical tumor suppressor. Loss of PTEN leads to the sustained accumulation of phosphatidylinositol (3,4,5)-trisphosphate (PI(3,4,5)P₃), which drives aberrant AKT activation and promotes tumor cell proliferation and survival [14]. The phosphoinositide phosphatase SHIP2 also dynamically modulates intracellular PI(3,4,5)P₃ levels, thereby directly regulating the activation of both the MAPK/ERK and PI3K/AKT signaling pathways in CESC cells [15]. Clinical evidence further demonstrates that elevated expression of phosphorylated AKT (p-AKT) is significantly associated with poor prognosis in cervical cancer patients, highlighting the substantial clinical and prognostic relevance of phosphoinositide metabolic dysregulation [16].

To identify PPM-related prognostic genes and assess their value in CESC, this study integrated transcriptomic data from public databases with single-cell RNA sequencing analysis. First, we implemented differential expression analysis on bulk transcriptomic data to identify a candidate set of phosphoinositide metabolism-related genes (PPM-RGs) and establish their prognostic significance at the tissue level [17]. Second, we applied single-cell analysis to elucidate the expression patterns of these genes across various cellular subtypes within the tumor microenvironment, thereby revealing their cell-type-specific regulatory functions [18, 19]. The findings not only provide a scientific foundation for personalized therapy but also establish a theoretical basis for targeting PPM pathways, thus contributing to precision medicine in CESC.

## Materials and methods

### Data collection

The GSE9750 dataset related to CESC was retrieved from the GEO (https://www.ncbi.nlm.nih.gov/geo/), which comprised only 32 CESC tissue samples (CESC group) and 24 normal cervical epithelium tissue samples (control group) for differential expression analysis. It provided paired transcriptomic data of cervical cancer and normal cervical epithelium, and was suitable for the preliminary screening of tumor‑related differentially expressed genes. The sequencing platform used was GPL96.

Meanwhile, the original dataset of CESC (also used as test set_0) was obtained from TCGA (http://cancergenome.nih.gov/), consisting of 265 CESC tissue samples that had complete clinical data and survival information. This original dataset was further arbitrarily assigned to a training set and test set_1 at a ratio of 7:3, where 186 samples were assigned as the training set for risk model construction and related analyses, and the remaining 79 samples served as test set_1 for internal validation of the risk model. The clinical information table was provided in Additional file 1. All data were downloaded on July 14, 2025. Subsequently, the single-cell dataset E-MTAB-11,948 was acquired from the ArrayExpress database (https://www.ebi.ac.uk/biostudies/arrayexpress). Only 3 CESC tissue samples (CESC group) and 2 control samples (control group) from this dataset were included. Although this dataset had a limited sample size, it provided the necessary resolution for exploring cell-type-specific expression patterns in the tumor microenvironment at the single-cell level, and was mainly used for single-cell analysis. In addition, based on a previous study [20], the gene set “REACTOME_PI_METABOLISM.v2023.2.Hs.gmt” was collected from the MSigDB (https://www.gsea-msigdb.org/gsea/msigdb), resulting in 84 PPM-RGs (Additional file 2).

For experimental validation, we selected cervical biopsy tissues from 32 patients with pathologically confirmed cervical cancer. The control group comprised tissues from ten patients who underwent hysterectomy for abnormal uterine bleeding. Written informed consent was obtained from all patients before surgery, authorizing the use of their samples for this research. Approval for the study protocol was granted by the Human Research Ethics Committee of the PLA General Hospital of the Central Theater Command. The clinicopathological information of all subjects was provided in Additional file 3.

### Acquisition of candidate genes

Differentially expressed genes (DEGs) between the CESC and control groups in GSE9750 dataset (32/24 samples) were characterized via “limma” package (v 3.54.0) with |log_2_FC| > 0.25 and p.adjust < 0.05 [21]. A volcano plot was yielded via “ggplot2” package (v 3.5.2) with the top 10 genes with upregulation and downregulation (ordered by |log_2_FC|) labeled [22]. A heatmap of these labeled genes was constructed using the “ComplexHeatmap” package (v 2.16.0) [23]. Then, “ggvenn” package (v 0.1.10) was used to perform an intersection analysis between DEGs and PPM-RGs [24], and candidate genes were determined from the overlapping genes.

For investigating the related functions and pathways of candidate genes, GO and KEGG enrichment analyses were conducted via “clusterProfiler” package (v 4.10.1) with a cutoff value of p.value < 0.05 [25]. Partial enrichment results were presented, ordered from high to low based on the number of enriched genes.

After obtaining the above candidate genes, to explore their interactions at the proteomic level, we imported them into the STRING database (http://string-db.org) with an interaction score threshold > 0.4, and constructed a protein–protein interaction (PPI) network.

### Determination of prognostic genes and risk models

In the training set, univariate Cox regression analysis was implemented relying on the aforementioned candidate genes via “survival” package (v 3.8-3) with the criteria of HR ≠ 1 and p.value < 0.05, to elucidate genes linked to prognosis (risk factors were defined as genes with HR > 1 [26], while protective factors were defined as those with HR < 1). A forest plot was drawn via “forestplot” package (v 2.0.1) to visualize the analysis results [27]. The PH assumption test was then conducted on the results of the univariate Cox regression analysis via cox.zph function (p.value > 0.05), and genes that fulfilled this test were designated as prognostic genes for subsequent analyses.

Additionally, an RSF model was first constructed for the prognostic genes via “randomForestSRC” package (v 3.2.3) and based on three datasets [28]: The training set, test set_0, and test set_1. Each patient’s risk score in the three datasets were computed using the predict function, with the formula defined as the average of the cumulative hazard function predictions for the patient across all survival trees, that is, $\:\mathrm{h}(t|{x}\mathrm{)=}\frac{\mathrm{1}}{{B}}{\sum\:}_{{i}\mathrm{=1}}^{{B}}{\mathrm{h}}_{{i}}\mathrm{(}t|{x}\mathrm{)}$ (where *hi(t|x)* denotes the predicted outcome of the i-th tree for patient x at time t, and *B* signifies the number of survival trees in the RSF model). Meanwhile, an importance score plot of the prognostic genes in the RSF model was generated for the training set.

Subsequently, we determined the optimal cutoff value for the risk score via “survminer” package (v 0.5.0) in the three datasets [29], and high-risk group (HRG) and low-risk group (LRG) were defined for samples in each dataset in light of this cutoff value. K-M curves (log-rank test, p.value < 0.05) via “survminer” package (v 0.5.0), 1-, 2-, and 3-year ROC curves via “survivalROC” package (v 1.0.3.1) [30], in the three datasets. Among them, when the AUC of the ROC curve was greater than 0.6, the model was considered to have credibility.

To further clarify the individual contribution of each prognostic gene to the risk model, RSF models containing only a single prognostic gene were separately constructed on the training set, with the same parameter settings as the full model (ntree = 1000, mtry = 2). The discriminative ability and prognostic stratification capacity of each single‑gene model and the full model were compared using Harrell’s C‑index, time‑dependent AUC at 1, 2, and 3 years, and Kaplan–Meier curves.

### Development of a nomogram model

In the training set, univariate Cox regression analysis (HR ≠ 1, p.value < 0.05) and PH assumption test (p.value > 0.05) were implemented via “survival” package (v 3.8-3), incorporating clinical features (T stage, Stage, Age, M stage, and N stage) and the risk score. Multivariate Cox regression analysis (HR ≠ 1, p.value < 0.05) and PH assumption test (p.value > 0.05) were further undertaken.

Based on the independent prognostic factors gained by above results, a nomogram was built via “rms” package (v 6.8-1) [31]. The 1-, 2-, and 3-year survival rates of patients were predicted using “points” (scores of individual independent prognostic factors) and “total points” (sum of scores of all independent prognostic factors). A calibration curve of the nomogram was plotted utilizing “rms” package (v 6.8-1) to evaluate its predictive performance.

### Analyses related to different risk groups

To explore discrepancies in biological processes between the two risk groups, differential gene analysis was implemented in the training set via “DESeq2” package (v 1.40.0) to recognize risk-related DEGs and their corresponding |log_2_FC| values [21]. These risk-related DEGs were ordered from high to low by |log_2_FC|. GSEA was conducted via “clusterProfiler” package (v 4.10.1) in light of the “c2.cp.kegg.v7.5.1.symbols.gmt” gene set from the MSigDB (https://www.gsea-msigdb.org/gsea/msigdb). The top 5 notably enriched results were presented with the criteria of |NES| > 1 and p.adjust < 0.05.

The xCell algorithm was applied to estimate the relative abundance of 64 cell types in the two groups of the training set. Distinctions in cell abundance between various risk groups were compared using the Wilcoxon test, and cells with significant differences were defined as differential cell types (p.value < 0.05). The “psych” package (v 2.2.9) was adopted to explore correlations between differential cells [32], and between prognostic genes and differential cells, with the thresholds of |cor| > 0.3 and p.value < 0.05.

The TIDE scoring method was implemented to calculate T cell exclusion scores, TIDE scores, and T cell dysfunction scores for the two groups in the training set. Distinctions in these scores between the groups were compared using Wilcoxon test (p.value < 0.05).

To analyze differences in somatic mutations between different risk groups, the mutation frequency of genes of the training set was calculated via “maftools” package (v 2.18.0) [33]. Waterfall plots of the 20 most highly mutated genes in each group were generated.

Data of tumor drugs were retrieved from the GDSC database (https://www.cancerrxgene.org/). The IC_50_ of each tumor sample in the training set was predicted utilizing “pRRophetic” package (v 0.5) [34]. And drugs with significant differences in IC_50_ between different risk groups were screened using Wilcoxon test (p.adjust < 0.05). Moreover, “psych” package (v 2.2.9) was further implemented to explore correlations between prognostic genes and these significantly different drugs (|cor| > 0.3, p.adjust < 0.05). The top 10 most significant drugs were presented, sorted by p.adjust.

In the training set, Wilcoxon test was adopted to compare immune checkpoints (ICs) expression between different risk groups (p.value < 0.05) [35]. Meanwhile, correlation was implemented between prognostic genes and significantly different ICs utilizing “psych” package (v 2.2.9) with |cor| > 0.3 and p.value < 0.05.

### Single-cell RNA analysis

To explore the cellular mechanisms of CESC, a series of analyses were performed on the E-MTAB-11,948 single-cell dataset via “Seurat” package (v 4.3.0) [36]. First, the PercentageFeatureSet function was invoked to calculate three indicators for all samples in the dataset: nCount_RNA (the number of gene expression counts detected per cell), nFeature_RNA (genes detected per cell), and percent.mt (the proportion of mitochondrial genes). Second, data were filtered relying on preset criteria, with specific thresholds of 35,000 > nCount_RNA > 200, 7,500 > nFeature_RNA > 200, and percent.mt < 20%, to exclude low-quality cells. After quality control was completed, the IntegrateData function was implemented to integrate the data, and then the Merge function was applied to combine the data of each sample.

Subsequently, the filtered data were normalized, and genes with high variability among cells were recognized, thereby screening out the top 2,000 genes. Afterwards, the data were scaled. Principal components (PCs) for clustering were determined. The JackStrawPlot function was implemented to compute the p-values of 1–50 PCs (PCs 1:50), and PCs with statistical significance were identified (p.value < 0.05); the Elbowplot function was implemented simultaneously to visualize the results.

Thereafter, unsupervised clustering analysis on the cells was adopted, and the RunTSNE function was adopted for clustering visualization (resolution = 0.4). Finally, cell annotation was completed based on the CellMarker database (http://117.50.127.228/CellMarker/) and published literature [37]. At the same time, functional enrichment analysis of each cell type was adopted utilizing “ReactomeGSA” package (v 3.20) [38], and partial results of significant enrichment were presented (p.value < 0.05).

Additionally, the Wilcoxon test was implemented to probe the expression of prognostic genes in various cells between the 2 groups, thereby identifying potential key cells. The “CellChat” package (v 1.6.1) was adopted to construct an intercellular signal transduction network [39]. Among the operations, the aggregateNet function was called to build an aggregated cell-cell communication network, and the number of interactions (reflecting communication frequency) and weight (reflecting communication intensity) between various cell types were calculated simultaneously. Additionally, “CellChat” package (v 1.6.1) was adopted again to identify the ligand-receptor pairing relationships mediating communication, thereby inferring potential interactions.

To analyze the differentiation trajectory of potential key cells, we performed further dimensionality reduction and clustering for the potential key cells. Then, “Monocle” package (v 2.26.0) was adopted to implement pseudotime analysis on the potential key cells [40], and the expression of prognostic genes during different stages was analyzed. Furthermore, DoRothEA algorithm was implemented to analyze the gene expression data of potential key cells. In light of the DoRothEA-derived transcription factor-target gene regulatory network, the activity of each transcription factor in potential key cells between different groups was calculated and compared, so as to reveal the inter-group differences in transcription factor activity.

### Assessment of phosphoinositide metabolism pathway activity and stability in monocytes

To directly link the prognostic genes with phosphoinositide metabolism activity at the single-cell level, all annotated monocytes were extracted from the E-MTAB-11,948 dataset. The PPM pathway score was calculated using the AddModuleScore function of Seurat, and the differences between the CESC group and the control group were compared. Meanwhile, considering the limited sample size of the single‑cell data, 100 bootstrap downsampling iterations were performed on the monocytes. In each iteration, an equal number of cells were randomly sampled, the mean difference in PPM scores between the two groups was calculated, and the proportion of iterations with a consistent direction of difference was recorded to evaluate the stability of the results.

### Experimental verification

For immunohistochemistry, After blocking with 10% normal goat serum for 1 h, sections were incubated overnight at 4 ℃ with primary antibodies against INPP5K (1:100; Proteintech, 15098-1-AP) or VAC14 (1:100; Proteintech, 15771-1-AP). Subsequently, they were administered with a biotinylated goat anti-rabbit IgG (1:400, Boster, BA-1054) for 2 h at ambient temperature, after which a 1 h incubation with ExtrAvidin Peroxidase. Positive staining was visualized using DAB and H2O2 in Tris–HCl buffer. The cervical sections were then counterstained with hematoxylin for 2 min. For negative controls, the primary antibodies were eliminated and substituted with normal IgG. All images were captured using a light microscope, and the results were analyzed with Saiviewer software.

H‑score (Histochemistry score) is a semiquantitative histological scoring method for immunohistochemical assessment. It converts the percentage of positive cells and their staining intensity into a numerical value that integrates both the depth and the proportion of positive staining. The H‑score is calculated as ∑(p_i_ × i), where i denotes the staining intensity category (0 = negative, no color; 1 = weak, pale yellow; 2 = moderate, brownish yellow; 3 = strong, brown) and p_i_ is the percentage of cells in category i. The final score ranges from 0 to 300, with higher values indicating stronger overall positivity (combining intensity and proportion).

Percentage of positive cells = (number of positive cells) / (total number of cells). This ratio reflects the quantity of positive cells of the same type and is suitable for samples with a relatively homogeneous cell population.

Spearman’s rank correlation was used to assess relationships between the four markers: positive cell rate for INPP5K, positive cell rate for VAC14, H score for INPP5K, and H score for VAC14, and both tumor stage and differentiation grade. Additionally, Mann–Whitney U tests were performed to compare marker levels between early (stage I–II, *n* = 23) and advanced (stage III–IV, *n* = 9) disease, and between well/moderately differentiated (*n* = 20) and moderately poorly/poorly differentiated (*n* = 12) groups. All statistical tests were two sided.

### Statistical analysis

All analyses were adopted utilizing R programming language (v 4.3.3). Cohort comparisons were made via Wilcoxon test with p.value < 0.05.

## Results

### Acquisition and analysis of candidate genes related to PPM in CESC

**Fig. 1:**
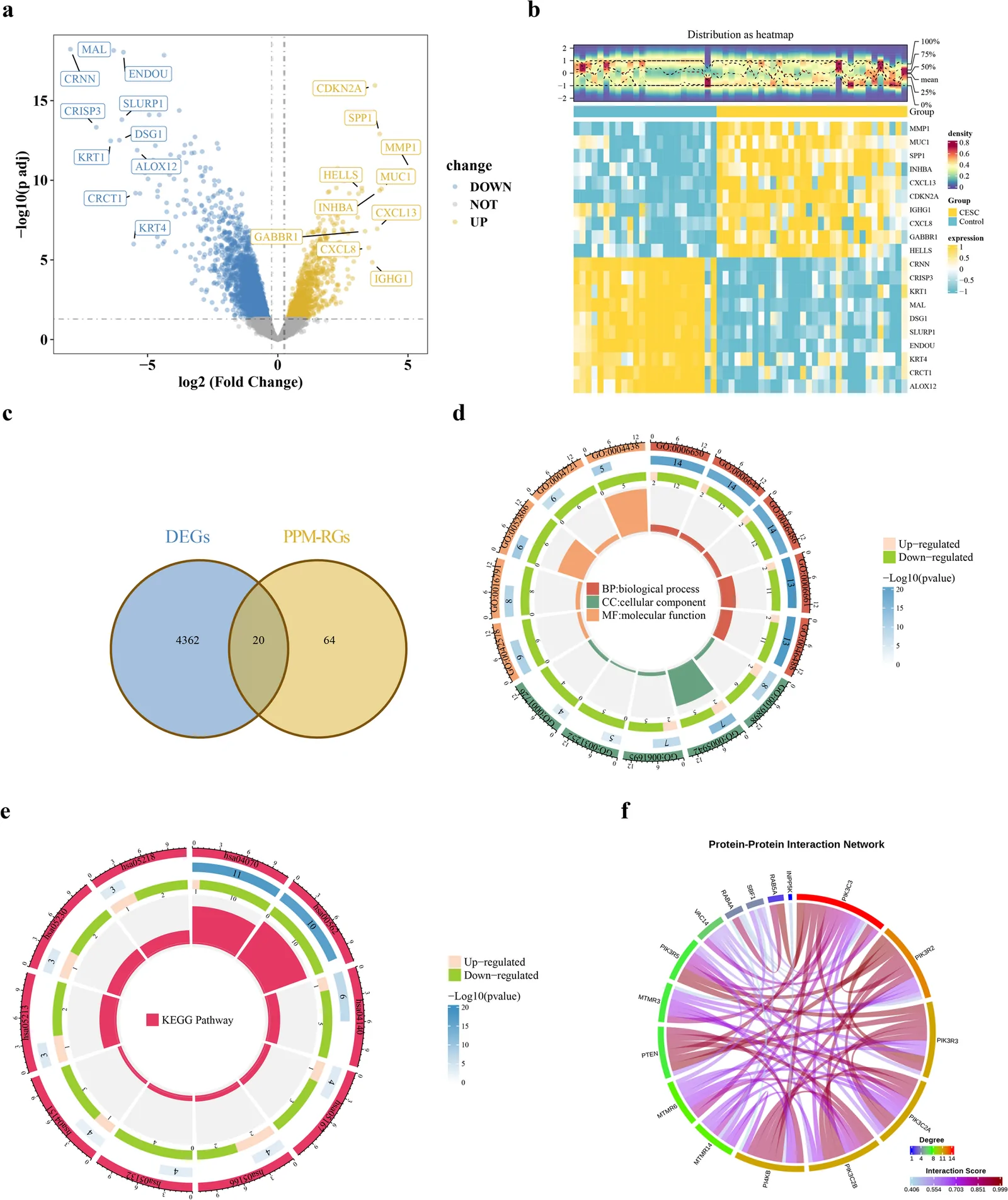
Screening and functional characterization of candidate PPM-related genes in CESC. **a** Volcano plot of differentially expressed genes between CESC and control samples in GSE9750, yellow represents up regulation, blue represents down regulation. **b **Heatmap of representative upregulated and downregulated genes. **c **Venn diagram showing the overlap between DEGs and PPM-RGs. **d **GO enrichment analysis of candidate genes. **e **KEGG enrichment analysis of candidate genes. **f **Protein-protein interaction network of candidate genes

Ultimately, 4,382 DEGs were screened from the GSE9750 dataset (32 cases/24 cases). Compared with the control group, 975 genes with upregulation and 3,407 genes with downregulation in the CESC group (Additional file 4). The volcano plot and heatmap of labeled genes of DEGs were exhibited in Figs. 1a-b, respectively. After that, the 20 candidate genes were obtained through intersection analysis (Fig. 1c; Additional file 5).

Further enrichment analysis was implemented on these 20 candidate genes (p.value < 0.05). The results exhibited that 333 functional items were gained by GO analysis, comprising 252 BP items, 33 CC items, and 48 MF items, involving multiple important functions such as glycerolipid metabolic process (GO:0046486), phosphatidylinositol biosynthetic process (GO:0006661), ruffle (GO:0001726), phosphoric ester hydrolase activity (GO:0042578), and phosphoprotein phosphatase activity (GO:0004721) (Fig. 1d; Additional file 6). KEGG enrichment analysis covered 95 pathways, such as phosphatidylinositol signaling system (hsa04070), inositol phosphate metabolism (hsa00562), autophagy-animal (hsa04140), and central carbon metabolism in cancer (hsa05230) (Fig. 1e; Additional file 7).

In addition, the PPI network constructed based on candidate genes showed that there were 62 interaction relationships. Notably, complex interaction patterns were exhibited among genes. For example, PIK3C3 could interact with RAB5A, PTEN, PIK3R2, and other genes. And PIK3C3 had the highest Degree value (Fig. 1f).

### Acquisition of prognostic genes and establishment of the risk model

**Fig. 2:**
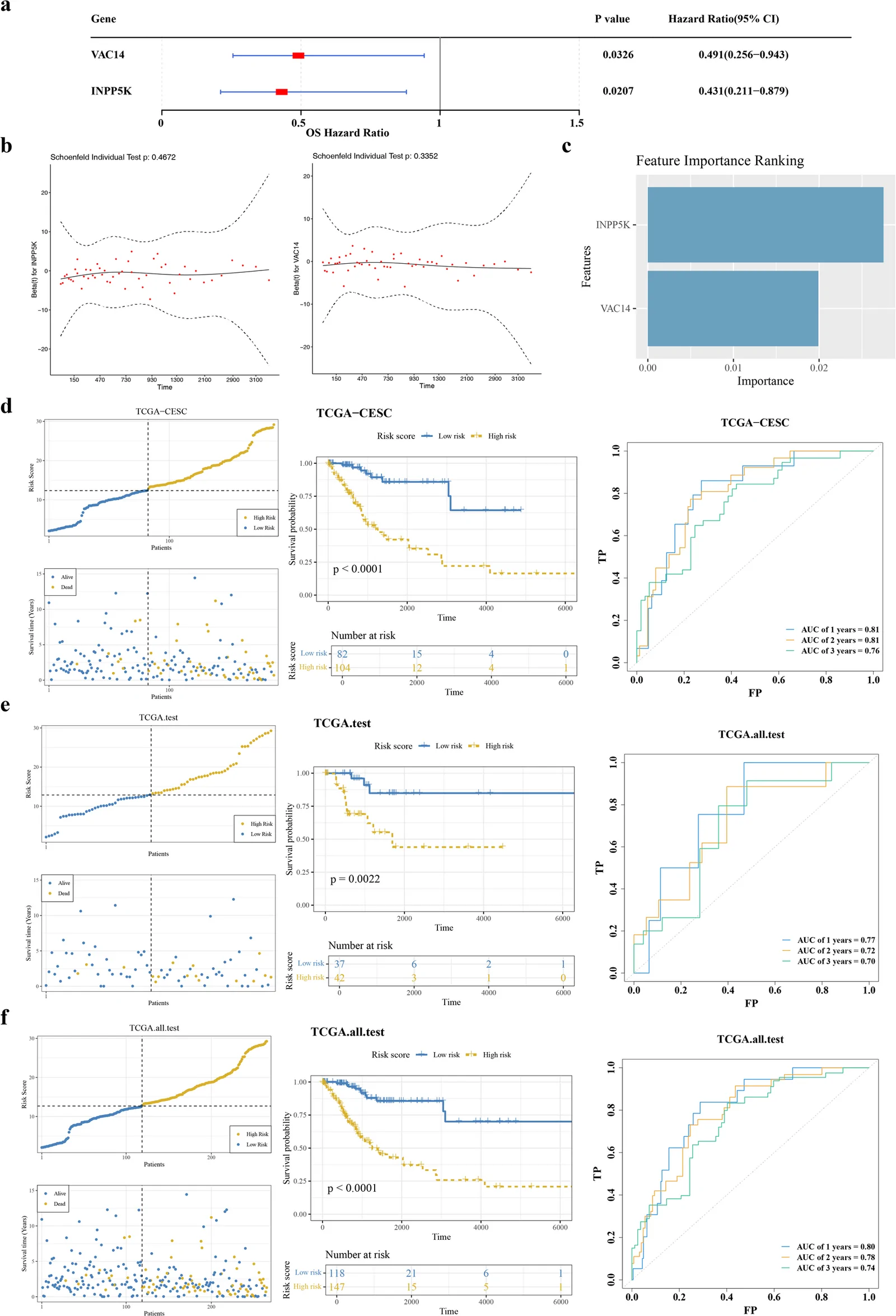
Identification of prognostic genes and construction of the risk model. **a **Forest plot of univariate Cox regression for candidate genes. **b **Proportional hazards assumption test plots for VAC14 and INPP5K. **c **Variable importance scores of prognostic genes in the RSF model. **d **Risk score distribution, survival status, Kaplan-Meier curve, and time-dependent ROC curves in the training set. **e **Risk score distribution, survival status, Kaplan-Meier curve, and time-dependent ROC curves in test set_1. **f **Risk score distribution, survival status, Kaplan-Meier curve, and time-dependent ROC curves in test set_0

Among the 20 candidate genes, the 2 prognostic genes (VAC14 and INPP5K) were identified, and both of them were protective factors (HR < 1). Specifically, these 2 genes were screened out by univariate Cox regression analysis (HR ≠ 1, p.value < 0.05) (Fig. 2a); further verified by PH assumption test, both of them met the test requirements (p.value > 0.05) (Fig. 2b). Overall, the risk model for predicting patients’ survival could be constructed, which was of great value for clinical decision-making.

When constructing the RSF model, ntree = 1000 and the maximum number of splitting nodes (mtry) = 2. Among the 2 prognostic genes, the importance score of INPP5K exceeded the importance score of VAC14 (Fig. 2c). Meanwhile, as determined by the optimal cutoff value 12.37026, the 186 samples in the training set were separated into a LRG (82 cases) and a HRG (104 cases); according to the optimal cutoff value of 12.92496, the 79 samples in test set_1 were separated into a HRG (42 cases) and a LRG (37 cases); similarly, relying on the optimal cutoff value of 12.73166, the 265 samples in test set_0 were separated into a HRG (147 cases) and a LRG (118 cases). Then, the related analyses based on the two groups divided by the three datasets presented that the K-M curves of the high- and low-risk groups were strikingly various (p.value < 0.05), and the survival rate of patients in the high-risk group was lower; the AUC values of 1-, 2-, and 3-years- ROC curves in the three datasets were all higher than 0.6 (training set: AUC = 0.81, 0.81, 0.76; test set_1: AUC = 0.77, 0.72, 0.70; test set_0: AUC = 0.80, 0.78, 0.74), suggesting that the model exhibited favorable predictive performance; and the survival status diagram and risk score curve further verified the practical applicability of the model (Figs. 2d-f).

To clarify the actual contribution of each prognostic gene to the model, we compared the performance of the full two‑gene model with that of the single‑gene models. The results showed that the full model achieved AUCs of 0.80, 0.80, and 0.74 at 1, 2, and 3 years, respectively, which were superior to those of any single‑gene model (Additional file 8), indicating that VAC14 and INPP5K had complementary value for the risk score and that retaining the two‑gene combination was a reasonable model choice.

### Nomogram model built by M stage and risk score

**Fig. 3:**
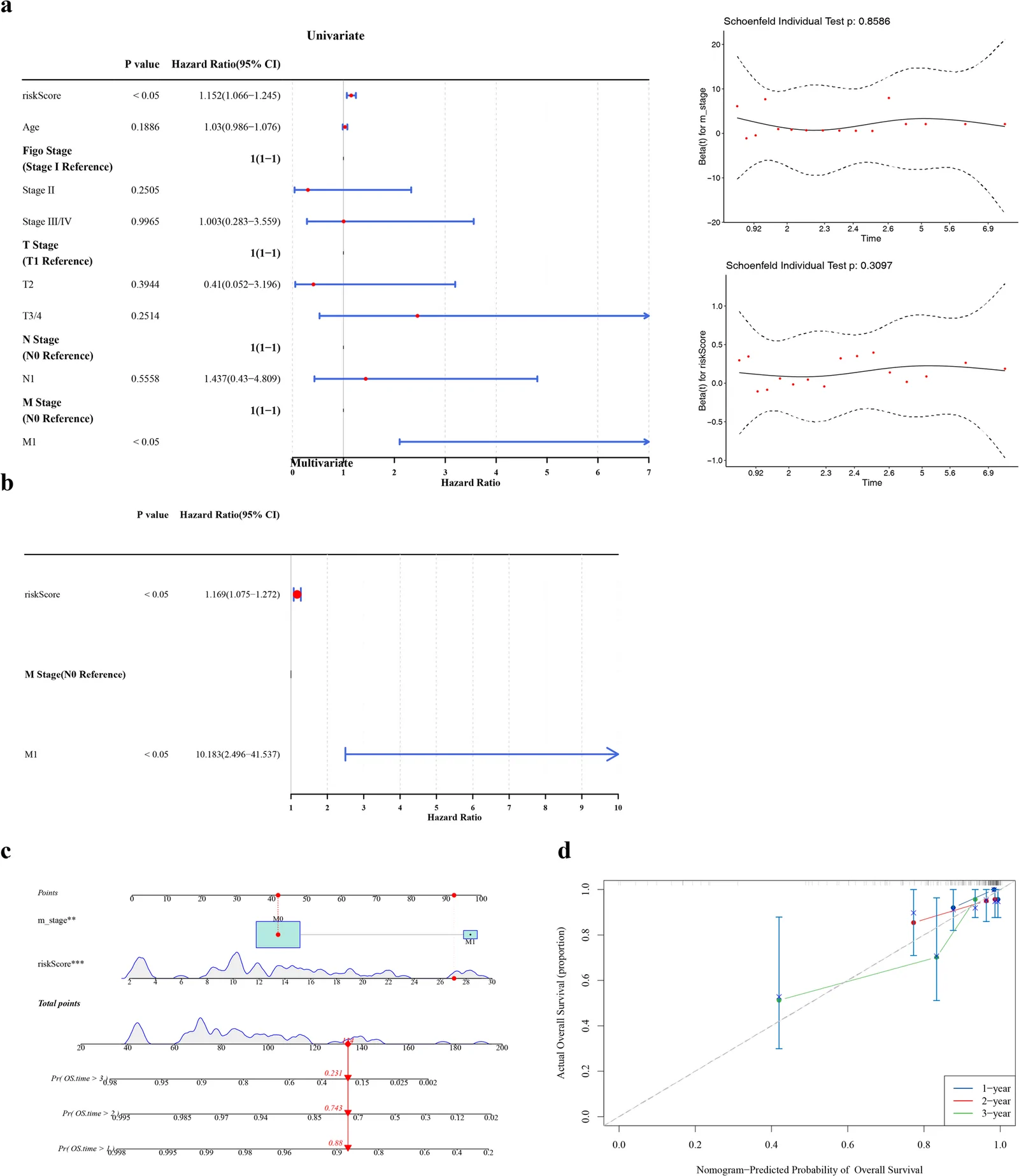
Development of the nomogram based on independent prognostic factors. **a **Regression analysis and proportional hazards assessment of candidate clinical factors and risk score. **b** Identification of independent prognostic factors. **c **Nomogram predicting 1-, 2-, and 3-year survival probabilities. **d **Calibration curves evaluating nomogram performance

After univariate/multivariate Cox regression analysis (HR ≠ 1, p.value < 0.05) and two PH assumption tests (p.value > 0.05), risk score and M stage were recognized as independent prognostic factors (Figs. 3a-b; Table 1). This was crucial for accurately predicting patients’ survival status and guiding treatment decisions.

**Table 1:** Results of multivariate PH assumption test

	chisq	df	*p* value
riskScore	0.9529	1	0.3290
m_stage	0.0001	1	0.9930

The nomogram was presented in Fig. 3c. When patients had an earlier M stage and a decreased risk score, they were usually more prone to have a longer survival period and a better survival status. And the calibration curve (Fig. 3d) showed that its slope was close to 1, and the predicted probabilities of 1-, 2-, and 3-year survival had largely overlapped with the reference line, indicating that the nomogram had good forecasting performance.

### Pathway enrichment, cell infiltration, and TIDE analysis between different risk groups

**Fig. 4:**
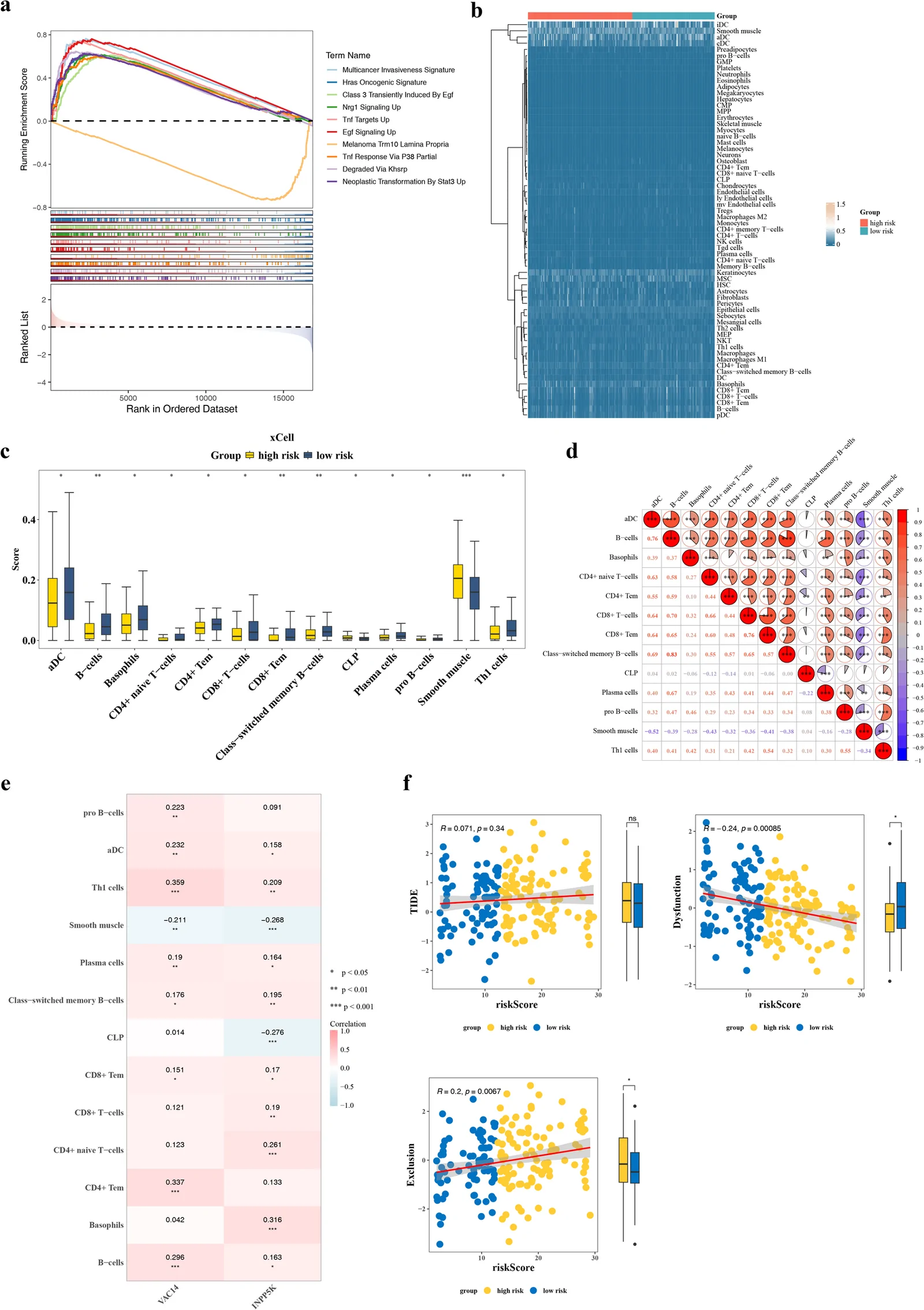
Pathway enrichment, immune infiltration, and TIDE analyses between risk groups. **a **Top significantly enriched pathways identified by GSEA. **b **Relative abundance of 64 infiltrating cell types in the two risk groups. **c **Differential cell types between the high-risk and low-risk groups, *p < 0.05, **p < 0.01, ***p < 0.001. **d **Correlation analysis among differential cell types, *p < 0.05, **p < 0.01, ***p < 0.001. **e **Correlations between prognostic genes and differential cell types, *p < 0.05, **p < 0.01, ***p < 0.001. **f **Comparison of TIDE-related scores between the two risk groups

In GSEA analysis, 1,597 significantly enriched pathways were obtained (|NES| > 1, p.adjust < 0.05; Additional file 9). As shown in Fig. 4a, the strikingly different pathways between two groups involved multicancer invasiveness signature, HRAS oncogenic signature, class 3 transiently induced by epidermal growth factor (EGF), neuregulin (NRG)1 signaling up, and tumor necrosis factor (TNF) targets up, etc.

Subsequently, in cell infiltration analysis, the relative abundances of 64 cell types in the two groups were displayed (Fig. 4b), and 13 differential cell types were identified between two risk groups (p.value < 0.05), including Activated dendritic cells (aDCs), B-cells, Basophils, etc. (Fig. 4c). And most of the differential cell types were correlated. Among them, Class-switched memory B-cells and B-cells were strikingly most positively linked (cor = 0.830, p.value < 0.05), and Smooth muscle and aDCs were strikingly most negatively linked (cor = − 0.520, p.value < 0.05) (Fig. 4d). While VAC14 was only strikingly positively correlated with T helper (Th)1 cells and CD+ effector memory T cells (Tem) (cor = 0.359, 0.337, p.value < 0.05); INPP5K was only strikingly positively linked with Basophils (cor = 0.316, p.value < 0.05) (Fig. 4e).

In TIDE analysis, the T cell exclusion scores were strikingly higher in the HRG than in the LRG (p.value = 0.00085, 0.0067); while there was no notable distinction in TIDE scores (p.value = 0.34) (Fig. 4f). The above results suggested that differences in gene-related pathways, cell infiltration, and T cell function might be closely related to risk grouping, and thus to the biological characteristics and prognosis of CESC patients.

### Somatic mutation, drug sensitivity, and ICs analysis between HRG and LRG

**Fig. 5:**
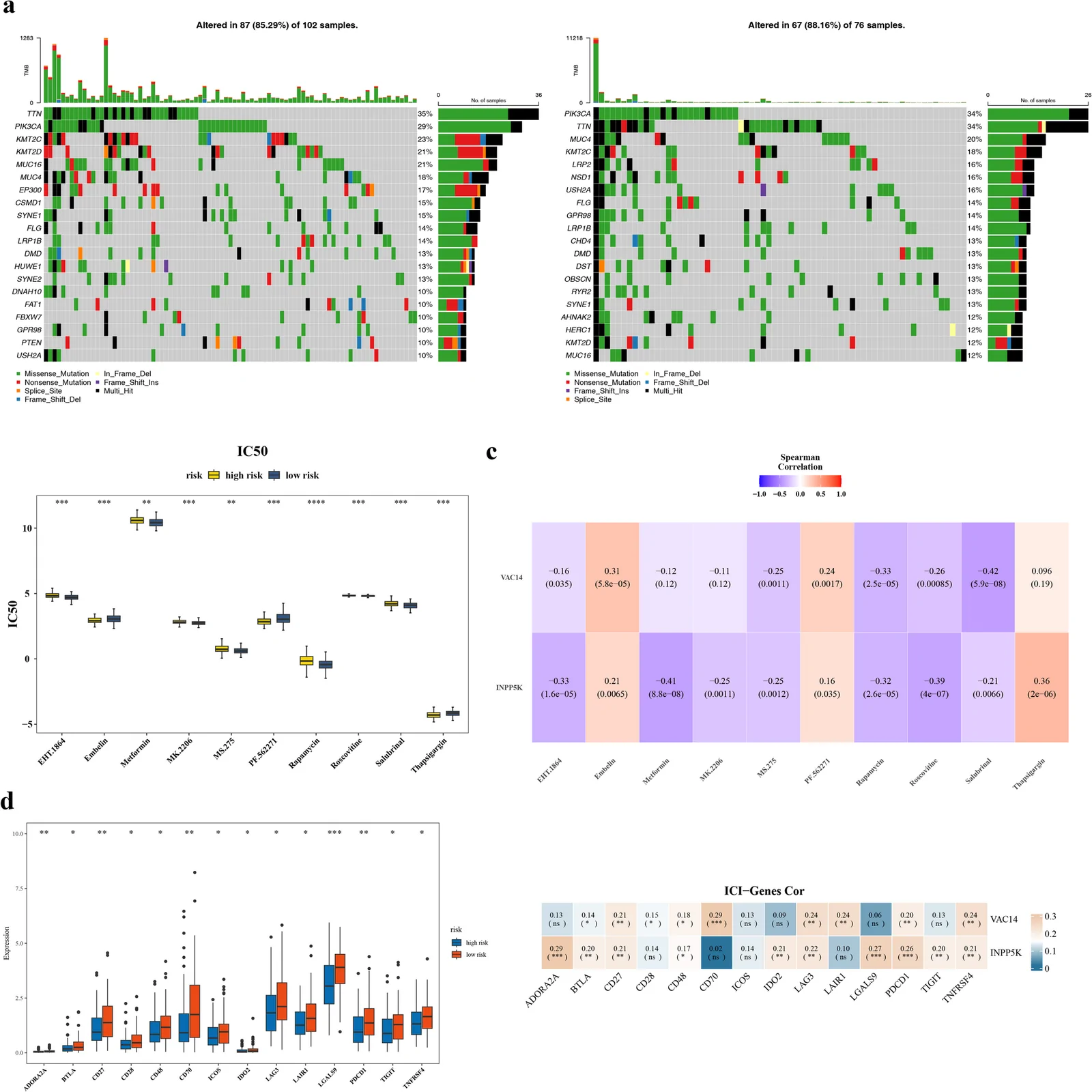
Somatic mutation, drug sensitivity, and immune checkpoint analyses in different risk groups. **a **Waterfall plots of the top somatic mutations in the high-risk and low-risk groups. **b **Top differential drugs ranked by adjusted P value, *p < 0.05, **p < 0.01, ***p < 0.001. **c **Correlations between prognostic genes and significantly different drugs. **d **Differential immune checkpoint expression and correlations with prognostic genes, *p < 0.05, **p < 0.01, ***p < 0.001

Then, in somatic mutation analysis, 87 out of 102 samples (85.29%) in the HRG possessed somatic mutations, while 67 out of 76 samples (88.16%) in the LRG possessed somatic mutations; the top three genes ranked by mutation frequencies in the HRG were TTN (35%), PIK3CA (29%), and KMT2C (23%), and in the LRG were PIK3CA (34%), TTN (34%), and MUC4 (20%), and the dominant mutation type in both groups was missense mutation (Fig. 5a). Notably, although there was an overlap of some high-frequency mutated genes, only some of the top 20 mutated genes in the two groups overlapped. The overall mutation frequency in the HRG was lower, and some genes had nonsense mutations as the main mutation type, which was different from that in the LRG.

Finally, in drug sensitivity analysis, it was found that the IC₅₀ of 15 drugs were strikingly distinct between the HRG and LRG (p.adjust < 0.05) (Additional file 10), and the top 10 remarkable drugs relying on p.adjust ranking included EHT.1864, Embelin, and Metformin, etc. (Fig. 5b). Among these 15 drugs, some drugs were strikingly linked with prognostic genes (|cor| > 0.3, p.adjust < 0.05; Additional file 11), and the correlation between the 10 most significant drugs and prognostic genes was also shown (Fig. 5c). It was worth noting that VAC14 was strikingly positively linked with Embelin (cor = 0.31, p.adjust = 5.8e-05) and most strikingly negatively linked with Salubrinal (cor = -0.42, p.adjust = 5.9e-08); INPP5K was strikingly positively linked with Thapsigargin (cor = 0.36, p.adjust = 2e-06) and most strikingly negatively linked with Metformin (cor = -0.41, p.adjust = 8.8e-08).

In addition, in ICs analysis, the 14 ICs were found to be strikingly divergent between HRG and LRG (p.value < 0.05), and among the 14 ICs, VAC14 had a weak positive link with CD70 (cor = 0.29, p.value < 0.05), while INPP5K had a weak positive correlation with ADORA2A (cor = 0.29, p.value < 0.05) (Fig. 5d). The above results suggested potential directions for tumor immunotherapy and personalized medicine; however, these were only predictive associations and still required further experimental verification.

### Annotation of 9 cell types and identification of potential key cells

**Fig. 6:**
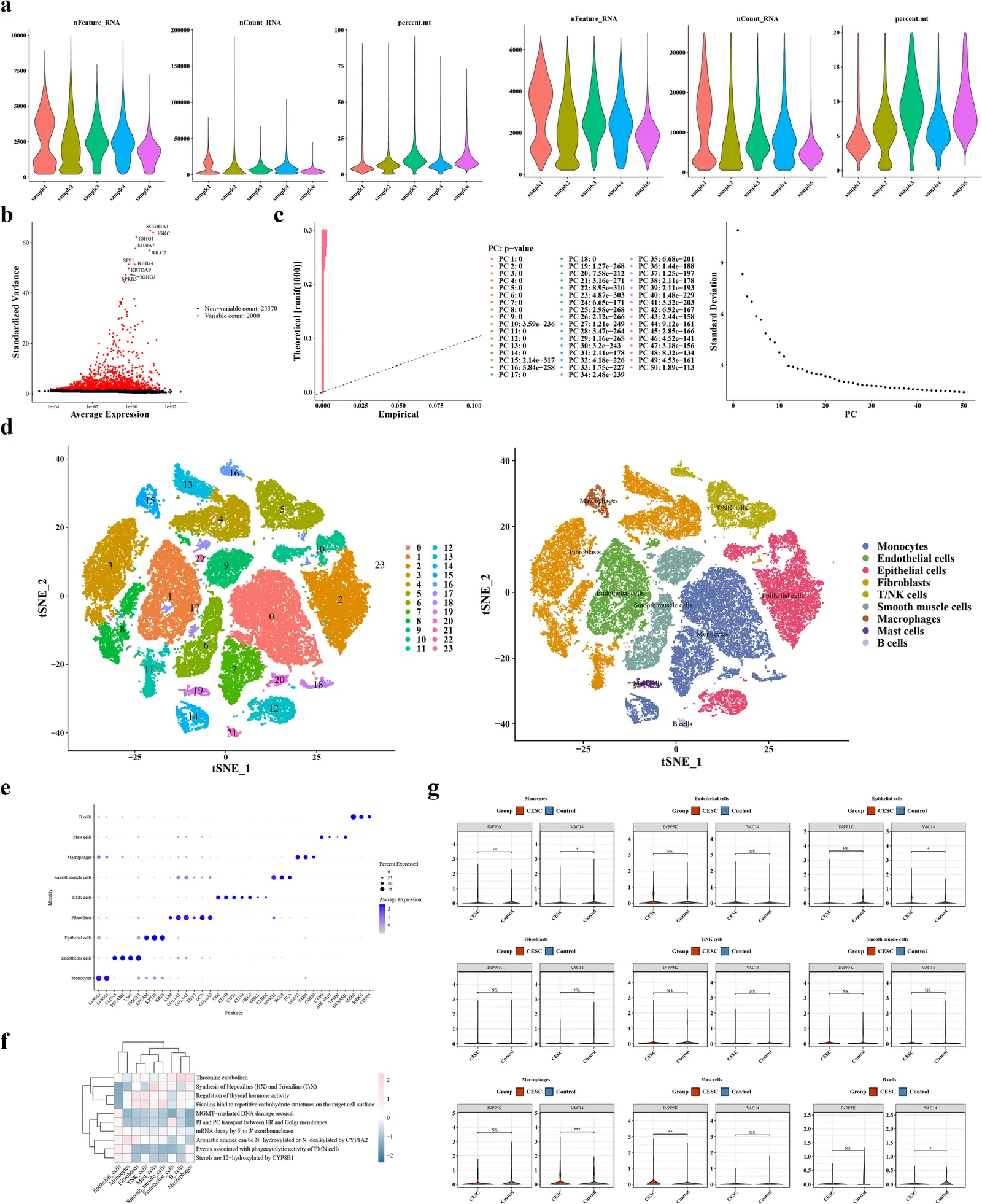
Single-cell landscape and identification of potential key cells in CESC. **a **Quality-control metrics before and after filtering. **b **Top 10 highly variable genes. **c **Elbow plot and JackStraw plot for principal component selection. **d **t-SNE plots showing cell clusters and annotated cell types. **e **Dot plot of marker genes for cell-type annotation. **f **Functional enrichment analysis of annotated cell types. **g **Differential expression of VAC14 and INPP5K across cell types, identifying monocytes as potential key cells, ns represents insignificant, *p < 0.05, **p < 0.01, ***p < 0.001

After bulk RNA analysis, single-cell RNA analysis was carried out based on the E-MTAB-11,948 single-cell dataset to further explore the potential mechanism of CESC at the cellular level. Sample data for subsequent analysis were obtained through data quality control. Through quality control, 59,959 cells were filtered to leave 52,063 cells, while the number of genes remained unchanged (27,370) (Fig. 6a). After data standardization, the 2,000 highly variable genes were screened for downstream analysis, and the top 10 genes with the highest variability were shown (Fig. 6b). Then, as shown in Fig. 6c, the actual p-values corresponding to different PCs were much smaller than the expected p-values, and the results were significant. At the same time, the first 30 PCs were subjected to ensuing analysis (p.value < 0.05). Subsequent clustering analysis identified 24 different cell clusters, and further annotated them into 9 cell types: Monocytes, Endothelial cells, Epithelial cells, T/Natural killer (NK) cells, Macrophages, Smooth muscle cells, Fibroblasts, Mast cells, and B cells (Fig. 6d; Table 2). The bubble chart showed that marker genes were highly specific, verifying the accuracy of the annotation (Fig. 6e).

**Table 2:** Marker genes used for cell annotation

Cluster	marker gene	Cell
0/7/14/18/20	S100A9、S100A8	Monocytes
1/17	CLDN5、PECAM1、VWF、TM4SF1	Endothelial cells
2/10/12/23	EPCAM、KRT18、KRT8	Epithelial cells
3/4/8/11/13	LUM、COL1A1、COL3A1、THY1、DCN、COL6A3	Fibroblasts
5/16	CD2、CD3D、CD3E、CD3G、NKG7、GNLY、KLRD1	T/NK cells
6/9/22	MYH11、RGS5、PLN	Smooth muscle cells
15	MS4A7、CD68、CD163	Macrophages
19	CTSG、ADCYAP1、TPSD1、GCSAML	Mast cells
21	MZB1、IGHG1、CD79A	B cells

Based on the obtained 9 cell types, functional enrichment analysis was performed (p.value < 0.05), and 1,706 pathways were enriched (Additional file 12), among which the top 10 pathways mainly involved 12-hydroxylation of sterols by CYP8B1, phosphatidylinositol (PI) and phosphatidylcholine (PC) transport between endoplasmic reticulum (ER) and Golgi membranes, regulation of thyroid hormone activity, synthesis of hepoxilins (HX) and trioxilins (TrX), and threonine catabolism, etc. (Fig. 6f). Meanwhile, through the Wilcoxon rank-sum test, it was found that prognostic genes were all significantly differentially expressed in Monocytes, so monocytes were recognized as potential key cells (Fig. 6g).

### Comprehensive analyses for potential key cells

**Fig. 7:**
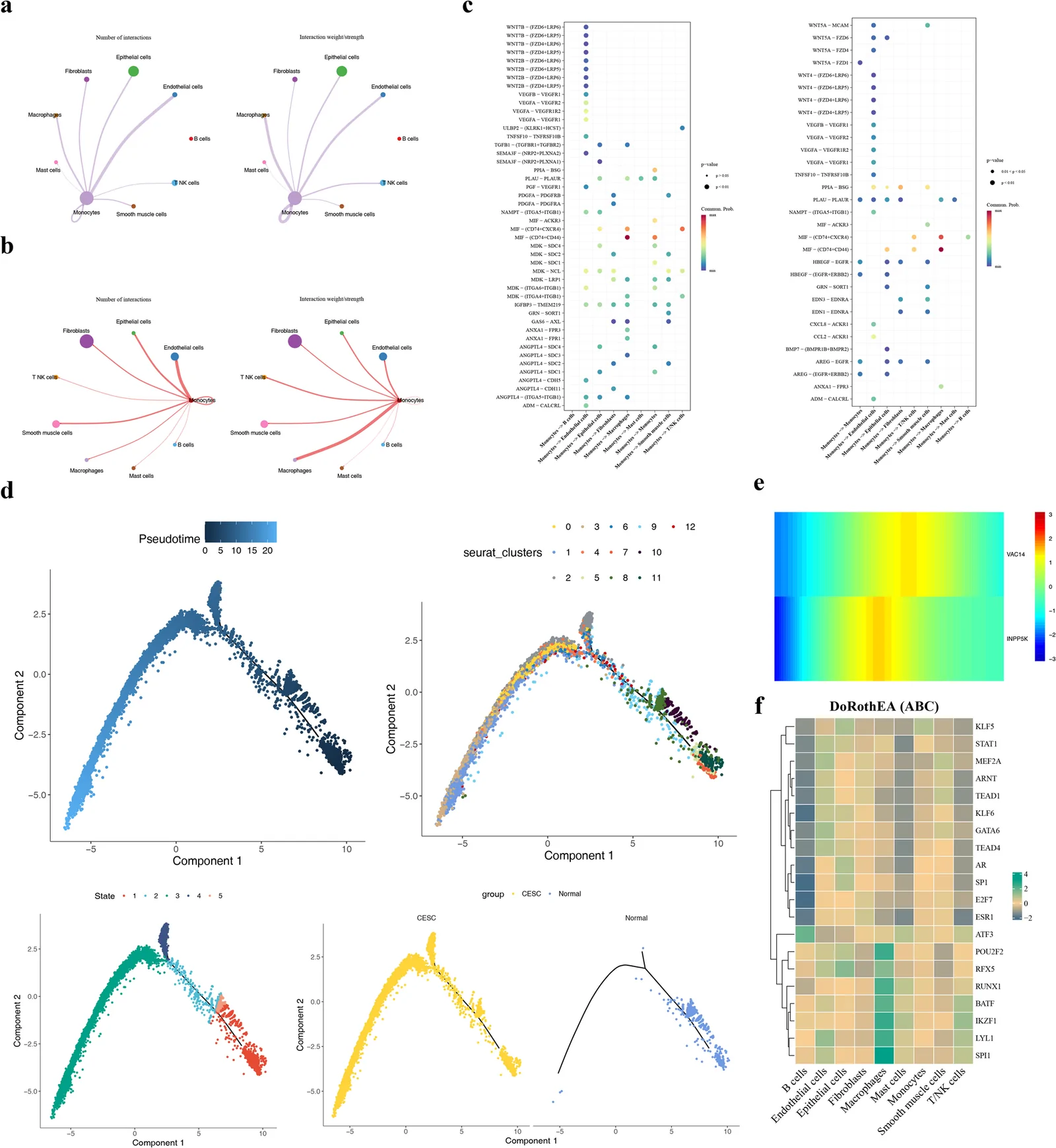
Intercellular communication, pseudotime trajectory, and transcription factor activity of monocytes. **a **Cell-cell communication network in the CESC group. **b **Cell-cell communication network in the control group. **c **Ligand-receptor interactions between monocytes and other cell types. **d **Pseudotime trajectory analysis of monocyte subpopulations. **e **Dynamic expression changes of VAC14 and INPP5K during monocyte development. **f **Thermogram of differential transcription factor activities across cell types

In terms of the cell communication network, remarkable distincts were shown between the CESC and control groups. In the CESC group, the weight and number of interactions between monocytes and other cells (such as Epithelial cells, Endothelial cells, etc.) were significantly changed, which was different from the interaction mode in the control group where Monocytes acted as the core interacting with Fibroblasts, Endothelial cells, etc. And the interaction characteristics of specific cell types such as B cells in the CESC group were also different from those in the control group (Figs. 7a-b). These results suggested that during the occurrence and development of CESC, the intercellular communication network might have been reconstructed, and the roles of potential key cells such as Monocytes in intercellular communication might have undergone important changes. Then, as presented in Fig. 7c, there were remarkable distincts in the ligand-receptor communication mode between Monocytes and other cells between the control group and the CESC group. Specifically, in the CESC group, the communication probability and significance (p-value < 0.01) of ligand-receptor pairs related to WNT family, MDK, etc. were different from those in the control group, and the direction of intercellular communication and the involved cell types also changed, suggesting that the cell communication mediated by these ligands and receptors might contribute to the tumorigenesis and progression of CESC.

After that, secondary dimensionality reduction analysis was performed on Monocytes, and the first 20 PCs (p.value < 0.05) were employed for successive clustering, and 13 cell subpopulations were determined (Additional file 13). The further pseudotime analysis results were shown in Fig. 7d. Monocytes evolved from the initial state to the terminal state along a specific path, revealing that there was a continuous state transition during their development. And these 13 cell subpopulations showed a specific spatial distribution pattern on the pseudotime trajectory, indicating that cells in different clusters were at different stages of development, reflecting the close relationship between cell heterogeneity and developmental timing. The distribution of each cell state on the trajectory showed a dynamic characteristic of gradually transitioning from one dominant state to the other states, suggesting that Monocytes underwent multiple intermediate stages during differentiation or functional remodeling. It was worth noting that there were remarkable variations in the distribution of Monocytes between the CESC group and the normal control group on this pseudotime trajectory, implying that the tumor microenvironment might interfere with or reconstruct the normal development process of Monocytes, thereby affecting their functional state. Meanwhile, Fig. 7e showed the expression trend changes of prognostic genes (VAC14, INPP5K) at different time stages. During the development of Monocytes, the expression levels of VAC14 and INPP5K both showed a trend of first increasing, reaching a peak in the middle stage, and then decreasing. The above results revealed that the intercellular communication network in CESC was reconstructed, the communication roles, ligand-receptor communication modes, and developmental processes of potential key cells such as Monocytes were all affected by the tumor microenvironment, and the prognostic genes VAC14 and INPP5K showed specific expression trends during the development of Monocytes.

In addition, as presented in Fig. 7f, remarkable variations in the activity of different transcription factors were observed among various cell types. For example, ATF3 was shown to exhibit relatively strong activating activity in B cells; POU2F2, RUNX1, and others were found to have prominent activating activity in Macrophages; while ESR1 was demonstrated to show inhibitory activity in some cells. These differences reflected the specificity of transcription factors in regulating gene expression in different cell types—a finding that was helpful for deeply understanding the molecular regulatory mechanisms of different cells during functional differentiation and disease occurrence.

### PPM pathway activity in monocytes and its stability validation

In monocytes, the PPM pathway score was significantly higher in CESC‑derived cells than in control cells (*p* = 0.00056, Additional file 14a), suggesting that the tumor microenvironment may have promoted the activation of phosphoinositide metabolism pathways in monocytes. The 100‑iteration bootstrap stability assessment showed that the direction of the mean difference in monocyte PPM scores between the CESC and control groups remained consistent, indicating a certain degree of robustness (Additional file 14b). However, given that the dataset included only 3 CESC and 2 control samples, these single‑cell findings should still be regarded as hypothesis‑generating and require independent validation in larger cohorts.

### Expression of VAC14 and INPP5K in CESC tissues

**Fig. 8:**
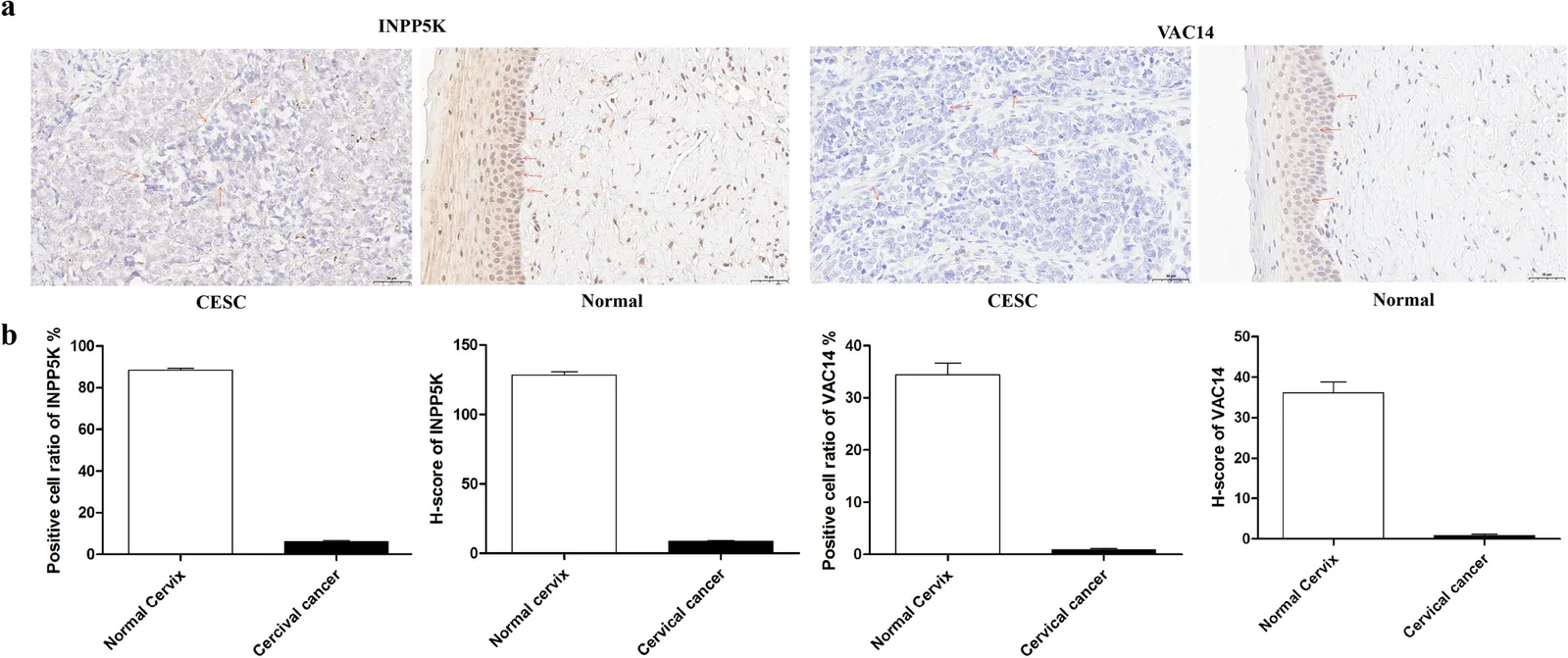
Immunohistochemical validation of VAC14 and INPP5K in CESC tissues. **a **Representative immunohistochemical images of INPP5K and VAC14 in normal and CESC tissues. **b** Quantitative comparison of positive cell rates and H-scores for INPP5K and VAC14

In this study, we further validated the expression of prognostic genes through immunohistochemical staining. In normal cervical tissues, INPP5K was strongly expressed in the nuclei of squamous epithelial, columnar epithelial, and vascular endothelial cells. VAC14, by contrast, exhibited weak cytoplasmic expression within the epithelial cells. In CESC tissues, INPP5K expression shifted to the cytoplasm and was significantly reduced. VAC14 expression was also dramatically downregulated, becoming nearly undetectable (Fig. 8a). Quantitative analysis confirmed the marked downregulation of both proteins in CESC. The positive cell rate for INPP5K declined from 88.46% ± 0.83% in normal epithelium (*n* = 10) to 6.08% ± 0.64% in cervical cancer epithelium (*n* = 32) (p.value < 0.0001). Its H-score similarly decreased from 128.4 ± 2.23 (*n* = 10) to 8.47 ± 0.77 (*n* = 32) (p.value < 0.0001). VAC14 exhibited an even more pronounced reduction, with its positive cell rate falling from 34.42% ± 2.25% (*n* = 10) to 0.86% ± 0.30% (*n* = 32) and its H-score from 36.17 ± 2.66 (*n* = 10) to 0.86 ± 0.30 (*n* = 32) (both p.value < 0.0001) (Fig. 8b). Consistent with the above intergroup comparison between normal cervical tissues and CESC tissues, further correlation analyses were performed to explore the associations of INPP5K and VAC14 protein expression with clinicopathological parameters including tumor stage and histological differentiation grade in the 32 cervical cancer specimens. All four markers showed significant negative correlations with stage (all *p* < 0.05), with INPP5K markers exhibiting stronger associations (|rho| > 0.5 for both rate and H score). Both INPP5K and VAC14 expression (positive cell rate and H score) correlated significantly and negatively with FIGO stage and tumour grade, suggesting that these proteins are downregulated in advanced and poorly differentiated disease. Notably, INPP5K showed stronger associations with both clinicopathological parameters than did VAC14. Furthermore, in normal cervical tissues (10 cases, data not shown), expression levels were markedly higher (e.g., mean INPP5K positive cell rate ~ 0.88 and mean H score ~ 128), supporting the notion that this pathway is suppressed during cervical carcinogenesis. These findings warrant further validation in larger cohorts and functional studies to clarify the mechanistic roles of INPP5K and VAC14 (Additional file 15).

## Discussion

Phosphatidylinositol and its phosphorylated derivatives, collectively known as phosphoinositides, represent a fundamental component of the intracellular signal transduction system, regulating virtually all aspects of cellular biology. Genes involved in phosphoinositide metabolism have been implicated in tumorigenesis; however, their precise roles in CESC remain poorly understood. In the research, relying on CESC datasets from public databases and 84 PPM-RGs, we identified 4,382 DEGs and subsequently narrowed down to 20 overlapping candidate genes. Univariate Cox regression analysis ultimately identified VAC14 and INPP5K as significant protective prognostic factors. The risk prediction model developed using RSF demonstrated robust performance (AUC > 0.7). Patients classified into the HRG displayed strikingly shorter overall survival, and both the M stage and risk score were recognized as independent prognostic factors. GSEA analysis revealed that the HRG was markedly enriched in pathways linked to malignant phenotypic regulation, accompanied by altered immune cell infiltration patterns and reduced immune scores. These findings suggest potential disruptions in intercellular communication and differentiation during CESC progression.

VAC14 and INPP5K are the two key protective prognostic genes identified in this study. Although both regulate the PPM network, their mechanisms differ. VAC14 is a highly conserved scaffold protein that forms a ternary complex with the lipid kinase PIKfyve and the phosphatase Fig. 4 to precisely regulate the dynamic balance of PI(3,5)P₂ on endosomal membranes, thereby controlling the maturation from early endosomes to late endosomes/lysosomes [41]. Proteomic analyses reveal that the PIKfyve–VAC14–Fig. 4 complex interacts with components of multiple cancer-associated pathways, including vesicle trafficking, PI3K/Akt signaling, and cell cycle regulation, suggesting VAC14 may maintain PI(3,5)P₂ homeostasis to modulate several tumor-related signaling networks [42]. INPP5K (also known as SKIP) is a phosphoinositide 5-phosphatase primarily localized to the endoplasmic reticulum, which hydrolyzes PI(4,5)P₂ and PI(3,4,5)P₃, thereby negatively regulating the activation intensity of the PI3K/Akt signaling pathway [43]. Notably, INPP5K resides in chromosomal region 17p13.3, a locus frequently deleted in various cancers. It has been proposed as a TP53-independent tumor suppressor, with its mRNA and protein expression significantly downregulated in endometrial cancer models [44]. In glioblastoma, the loss of INPP5K can lead to enhanced cell viability, reduced apoptosis, and a significant increase in tumorigenic capacity in vivo [45]. Consistent with this evidence, quantitative analysis of IHC staining in this study confirmed that both VAC14 and INPP5K exhibit significant protein downregulation in CESC tissues. The VAC14-positive cell rate dropped from 35.41% to 0.35%, while that for INPP5K decreased from 88.81% to 6.13%, indicating both may serve important tumor-suppressive roles in CESC. Furthermore, INPP5K localizes to the nucleus in normal cervical tissues but shifts to the cytoplasm in cancerous tissues. This altered subcellular distribution may affect its hydrolysis efficiency toward PI(4,5)P₂, a mechanism that requires further study [44]. In summary, the marked protein downregulation and potential functional alterations of VAC14 and INPP5K in CESC suggest they may jointly disrupt phosphoinositide metabolic homeostasis, enabling aberrant activation of downstream oncogenic signals and promoting cervical cancer progression. Within the dysregulated signaling networks of tumors, altered expression of VAC14 and INPP5K may be associated with the aberrant activation of oncogenic cascades. The HRG exhibited significant enrichment in classical oncogenic pathways, including the “HRAS oncogenic signature” and “STAT3-mediated neoplastic transformation”. These pathways are critically involved in driving malignant phenotypes, such as aberrant proliferation, evasion of apoptosis, and enhanced invasion and metastasis [46, 47]. HRAS mutation or hyperactivation drives cell-cycle dysregulation through sustained stimulation of the downstream RAF–MEK–ERK cascade [48], whereas persistent STAT3 activation transcriptionally upregulates anti-apoptotic effectors (e.g., Bcl-2) and promotes epithelial–mesenchymal transition (EMT) [49]. As PPM-associated genes, VAC14 and INPP5K likely exert indirect tumor-suppressive effects by modulating the metabolic balance of phosphoinositide species. As a key second messenger, PI(3,4,5)P₃ activates effectors including AKT, thereby indirectly influencing pathways such as HRAS and STAT3. INPP5K) negatively regulates this process by hydrolyzing PI(3,4,5)P₃, whereas VAC14 maintains PI(3,5)P₂ homeostasis to modulate lysosomal degradation. The coordinated action of INPP5K and VAC14 may thus suppress excessive activation of downstream HRAS and STAT3 signaling [50]. Pathways additionally enriched in the HRG like “upregulation of TNF target genes” and “activation of the EGF signaling pathway”, were also linked to reduced VAC14 and INPP5K expression, further supporting their role as negative regulators within the PPM network.

Beyond serving as prognostic biomarkers in CESC, the associations of VAC14 and INPP5K with specific therapeutic agents form a foundation for personalized treatment.

VAC14 expression showed a negative correlation with sensitivity to Salubrinal, an inhibitor of endoplasmic reticulum (ER) stress [51], suggesting that patients with higher VAC14 levels may exhibit enhanced responsiveness to Salubrinal. Salubrinal could therefore serve as a potential therapeutic option for this patient subgroup, although the underlying mechanisms require further validation. On the other hand, INPP5K expression positively correlates with sensitivity to the calcium modulator Thapsigargin [52, 53], indicating its potential utility as a predictive biomarker for Thapsigargin efficacy. Elevated INPP5K levels may sensitize cells to perturbations in intracellular calcium signaling, thereby enhancing the cytotoxic effects of Thapsigargin. Furthermore, the correlations between these two genes and immune checkpoint genes lay a theoretical foundation for combination immunotherapy, suggesting that modulating the expression of VAC14 or INPP5K may potentiate the therapeutic efficacy of immune checkpoint inhibitors. This hypothesis is also consistent with the current clinical advances in immunotherapy for recurrent or metastatic cervical cancer. PD‑1/PD‑L1 inhibitors, represented by pembrolizumab, have been approved for the treatment of advanced cervical cancer with positive PD‑L1 expression [54], and emerging strategies such as bispecific antibodies and adoptive cell therapy are also being actively explored [55, 56].These advancements highlight the potential of immune regulation in the treatment of cervical cancer, and this study further suggests that targeting VAC14 or INPP5K in combination with existing immune checkpoint inhibitors may have synergistic value, which is in line with this development trend to a certain extent. Admittedly, this hypothesis still requires systematic in vitro and in vivo functional assays as well as preclinical models for further validation.

In the cellular interaction network, monocytes consistently occupy a central position. Within the CESC group, however, the interactions between monocytes and endothelial or epithelial cells differ in both weight and strength from those in the control group. Cell communication analysis reveals more pronounced interaction signals between monocytes and endothelial cells in CESC, suggesting more frequent and robust crosstalk. This enhanced communication may be closely linked to processes like angiogenesis and immune cell infiltration in the tumor microenvironment. Conversely, interactions between monocytes and fibroblasts are more prominent in the control group, reflecting a distinct pattern of monocyte-mediated communication in normal versus tumor tissues. Ligand-receptor analysis further revealed distinct distributions and significance of pathway-related interactions in CESC compared to controls, notably within the WNT family (e.g., WNT7B-FZD) and VEGF family (e.g., VEGFA/B with their receptors). The WNT pathway is a classical regulatory pathway for cell proliferation and differentiation in tumorigenesis [57, 58]. Notably, the WNT7B-FZD ligand-receptor pair primarily mediates communication between monocytes and endothelial cells, suggesting that monocyte-derived WNT7B may promote endothelial cell activation in a paracrine manner and participate in tumor vascular remodeling [59, 60] Similarly, the interaction activity of the VEGF pathway is also elevated in CESC [61, 62]. These two pathways likely function synergistically during tumor angiogenesis rather than independently. In phosphoinositide metabolism, PI3K/Akt signaling phosphorylates GSK-3β to inhibit β-catenin degradation, thereby indirectly enhancing the transcription of WNT target genes. VEGF receptor downstream signaling also depends on PI3K/Akt activation [63].Consequently, the phosphoinositide metabolic disruption caused by VAC14 and INPP5K downregulation may amplify the pro-tumor effects of both WNT and VEGF signaling, establishing a positive feedback loop. Although similar interactions occur in the control group, their overall pattern reflects homeostatic regulation, contrasting sharply with the pro-tumor activation state in CESC. These findings imply that combining anti-angiogenic therapy with WNT pathway inhibition may have clinical potential for high-risk CESC patients, though further validation is necessary.

The interaction profiles of immune cells, including macrophages and mast cells, also differed markedly between groups. In the CESC group, interactions between monocytes and macrophages were skewed toward promoting tumor immune evasion or sustaining a chronic inflammatory state, thereby facilitating tumor progression. In contrast, similar interactions in the control group likely maintain tissue immune homeostasis and normal defensive functions. For instance, the CESC group likely exhibited a higher abundance of ligand-receptor signals that drive macrophage polarization toward pro‑tumor phenotypes (e.g., M2‑like), whereas signals in controls predominantly contributed to maintaining a balanced or homeostatic macrophage phenotype.

VAC14 and INPP5K displayed peak expression levels during the intermediate stage of monocyte development, implicating their potential involvement in modulating critical checkpoints of monocyte differentiation and maturation. Monocytes serve as pivotal mediators in antitumor immunity. They function not only as precursors to phagocytic and cytotoxic macrophages but also as antigen-presenting cells that initiate adaptive immune responses [64, 65]. Their elevated expression at the mid-stage of monocyte development may regulate monocyte differentiation and functional maturation by modulating downstream events in the PPM pathway, such as the phosphorylation status of inositol phospholipids. This regulatory role may help prevent polarization toward pro-tumorigenic phenotypes, thereby contributing to the maintenance of homeostasis in the tumor immune microenvironment [66].

However, this study has several inherent limitations. At the data level, the limited sample sizes for differential gene screening, single-cell analysis, and IHC validation may restrict the generalizability and robustness of our findings. Additionally, the IHC experiments were performed without independent blinded scoring, which may introduce observer bias and limit the generalizability of our observations. Although bootstrap methods were applied to assess the stability of single-cell analyses, the inferences regarding cell-type-specific functions and cell communication network reconstruction still require validation in larger cohorts. Accordingly, the relevant conclusions of this study should be considered hypothesis-generating rather than definitive. In terms of model development, this study lacks external validation based on independent test cohorts, which may potentially overestimate model performance. Future studies will incorporate independent large-scale datasets to enhance the robustness of both differential gene screening and model validation procedures. Furthermore, the current study only identifies correlative associations between VAC14/INPP5K expression and cervical cancer prognosis. Without direct in vitro functional experimental validation, we cannot establish definitive causal relationships between these core genes and malignant cellular phenotypes or the activity of the downstream PI3K/AKT pathway. Moreover, multiple analyses including GSEA, immune infiltration evaluation, and drug sensitivity prediction are predominantly based on inter-group comparative analyses, lacking in-depth mechanistic integration with core gene expression. Therefore, the inferred activation of oncogenic pathways remains speculative. Drug sensitivity outcomes are merely computational predictions and should not be regarded as definitive experimental evidence.

To address the above limitations, future research will expand sample sizes across all experimental and analytical procedures, repeat differential gene screening using independent large-scale datasets, and validate our findings via external independent cohorts and multi-center single-cell sequencing data. In addition, we will conduct systematic siRNA knockdown and gene overexpression experiments to detect cervical cancer cell proliferation, migration, apoptosis, and phosphorylation levels of the PI3K/AKT pathway. Candidate drugs will also be functionally validated through CCK-8 assays. These efforts will transform correlative observational results into solid causal evidence, providing a more rigorous experimental basis for targeted therapeutic strategies against phosphoinositide metabolism in cervical cancer.

## Conclusion

In summary, this research elucidates the putative molecular mechanisms linked to PPM in CESC, identifies prognostic genes, and constructs a risk model, thereby providing new perspectives into the pathogenesis of CESC and PPM-related research. In contrast, the clinical translation of these findings requires further validation through prospective data, and the precise molecular mechanisms involved warrant deeper investigation via subsequent experiments. Moving forward, advances in multi‑omics integration and functional studies are expected to expand and refine this research, ultimately contributing to a more solid theoretical foundation for early warning and precision therapy of CESC‑PPM.

## Supplementary Information

Supplementary Material 1: Additional file 1. Clinical Information of the Dataset Samples. Additional file 2. List of phosphoinositide metabolism-related genes (PPM-RGs) obtained from MSigDB. Additional file 3. List of clinical sample information. Additional file 4. Differentially expressed genes identified in GSE9750. Additional file 5. Overlapping candidate genes between DEGs and PPM-RGs. Additional file 6. GO enrichment results of candidate genes. Additional file 7. KEGG enrichment results of candidate genes. Additional file 8. Tim-dependent ROC curves at 1, 2, and 3 years for the full model (VAC14 + INPP5K), the VAC14-only model, and the INPP5K-only model. Additional file 9. Significantly enriched GSEA results between the high-risk and low-risk groups. Additional file 10. Differential drug sensitivity results between the high-risk and low-risk groups. Additional file 11. Correlations between prognostic genes and significantly different drugs. Additional file 12. Functional enrichment results of annotated cell types. Additional file 13. Monocyte subpopulation clustering and PCA dimensionality-reduction analyses. Additional file 14. PPM pathway activity in monocytes and bootstrap stability assessment. (a) Comparison of the PPM pathway score between CESC-derived and control-derived monocytes. (b) Bootstrap stability assessment of the mean difference in monocyte PPM scores between the CESC and control groups. Additional file 15 Correlative Analysis of INPP5K and VAC14 Expression with tumor stage (Supplementary Table 1) and differentiation grade (Supplementary Table 2).

## Data Availability

The data used in this study are publicly accessible from The Cancer Genome Atlas (TCGA), Gene Expression Omnibus (GEO), ArrayExpress and Molecular Signatures Database (MSigDB).

## Abbreviations

AKT: Protein kinase B
AUC: Area under the curve
CESC: Cervical cancer
DEGs: Differentially expressed genes
EMT: Epithelial-mesenchymal transition
ER: Endoplasmic reticulum
ERK: Extracellular signal-regulated kinase
GDSC: Genomics of Drug Sensitivity in Cancer
GEO: Gene Expression Omnibus
GO: Gene Ontology
GSEA: Gene set enrichment analysis
HR: Hazard ratio
HRG: High-risk group
HPV: Human papillomavirus
IC50: Half-maximal inhibitory concentration
ICs: Immune checkpoints
KEGG: Kyoto Encyclopedia of Genes and Genomes
LRG: Low-risk group
NK: Natural killer
PCs: Principal components
PH: Proportional hazards
PI3K: Phosphoinositide 3-kinase
PPI: Protein-protein interaction
PPM: Phosphoinositide metabolism
PPM-RGs: Phosphoinositide metabolism-related genes
PTEN: Phosphatase and tensin homolog
ROC: Receiver operating characteristic
RSF: Random survival forest
TCGA: The Cancer Genome Atlas
TIDE: Tumor Immune Dysfunction and Exclusion
