# Supplementary material for: Phosphoinositide metabolism-related prognostic genes and their prognostic value in cervical cancer: an investigation based on transcriptome, single-cell analyses and verification
Source: BMC Cancer. 2026 Jul 9;26:1180. doi: 10.1186/s12885-026-16509-x (PMC13632353; doi:10.1186/s12885-026-16509-x)
Supplement: Supplementary file 1 [file 12885_2026_16509_MOESM1_ESM.zip › Additional file 15.docx]

**Supplementary Table1. Correlation with tumor stage**

| **Marker** | **Spearman’s rho** | ***P* value** | **Early (I–II) mean ± SD** | **Advanced (III–IV) mean ± SD** | ***P* value*** |
| --- | --- | --- | --- | --- | --- |
| INPP5K positive cell rate | –0.612 | <0.001 | 0.0794 ± 0.0281 | 0.0133 ± 0.0069 | <0.001 |
| VAC14 positive cell rate | –0.418 | 0.017 | 0.0347 ± 0.0214 | 0 | 0.002 |
| INPP5K H score | –0.589 | <0.001 | 10.21 ± 3.56 | 3.12 ± 1.08 | <0.001 |
| VAC14 H score | –0.405 | 0.021 | 3.12 ± 2.11 | 0 | 0.003 |
| *P value from Mann–Whitney U test. | | | | | |

**Supplementary Table 2. Correlation with differentiation grade**

| **Marker** | **Spearman’s rho** | ***P* value** | **Well/Moderate (n=20) mean ± SD** | **Mod poor/Poor (n=12) mean ± SD** | ***P* value*** |
| --- | --- | --- | --- | --- | --- |
| INPP5K positive cell rate | –0.447 | 0.010 | 0.0744 ± 0.0284 | 0.0382 ± 0.0387 | 0.008 |
| VAC14 positive cell rate | –0.391 | 0.027 | 0.0322 ± 0.0198 | 0.0046 ± 0.0158 | 0.015 |
| INPP5K H score | –0.436 | 0.012 | 10.01 ± 3.42 | 5.23 ± 4.12 | 0.009 |
| VAC14 H score | –0.385 | 0.029 | 2.98 ± 2.05 | 0.45 ± 1.42 | 0.018 |
| *P value from Mann–Whitney U test. | | | | | |
